# CD105 expression in cancer-associated fibroblasts: a biomarker for bone metastasis in early invasive ductal breast cancer patients

**DOI:** 10.3389/fcell.2023.1250869

**Published:** 2023-08-31

**Authors:** María Belén Giorello, Leandro Marcelo Martinez, Francisco Raúl Borzone, María del Rosario Padin, María Florencia Mora, Ina Sevic, Laura Alaniz, María de Luján Calcagno, Hernán García-Rivello, Alejandra Wernicke, Vivian Labovsky, Norma Alejandra Chasseing

**Affiliations:** ^1^ Laboratorio de Inmunohematología, Instituto de Biología y Medicina Experimental (IBYME), Consejo Nacional de Investigaciones Científicas y Técnicas (CONICET), Buenos Aires, Argentina; ^2^ Division of Hematology and Medical Oncology, Department of Medicine, Weill Cornell Medical College, New York, NY, United States; ^3^ Departamento de Anatomía Patológica, Hospital Italiano, Buenos Aires, Argentina; ^4^ Laboratorio de Microambiente Tumoral, Centro de Investigaciones Basicas y Aplicadas (CIBA), Junín, Argentina; ^5^ Facultad de Farmacia y Bioquímica, Universidad de Buenos Aires, Buenos Aires, Argentina

**Keywords:** cancer-associated fibroblasts, CD105, breast cancer, prognosis factor, bone metastasis

## Abstract

**Introduction:** Bone metastasis is one of the causes that mainly decrease survival in patients with advanced breast cancer. Therefore, it is essential to find prognostic markers for the occurrence of this type of metastasis during the early stage of the disease. Currently, cancer-associated fibroblasts, which represent 80% of the fibroblasts present in the tumor microenvironment, are an interesting target for studying new biomarkers and developing alternative therapies. This study evaluated the prognostic significance of the CD105 expression in cancer-associated fibroblasts in early breast cancer patients.

**Methods:** Immunohistochemistry was used to assess CD105 expression in invasive ductal breast carcinomas (n = 342), analyzing its association with clinical and pathological characteristics.

**Results:** High CD105 expression in cancer-associated fibroblasts was associated with an increased risk of metastatic occurrence (*p* = 0.0003), particularly bone metastasis (*p* = 0.0005). Furthermore, high CD105 expression was associated with shorter metastasis-free survival, bone metastasis-free survival, and overall survival (*p* = 0.0002, 0.0006, and 0.0002, respectively). CD105 expression also constituted an independent prognostic factor for metastasis-free survival, bone metastasis-free survival, and overall survival (*p* = 0.0003, 0.0006, and 0.0001, respectively).

**Discussion:** The high CD105 expression in cancer-associated fibroblasts is an independent prognostic marker for bone metastasis in early breast cancer patients. Therefore, the evaluation of CD105(+) CAFs could be crucial to stratify BCPs based on their individual risk profile for the development of BM, enhancing treatment strategies and outcomes.

## 1 Introduction

Breast cancer is the most prevalent type of tumor in women, affecting a significant number of them worldwide (Meer et al., 2021). Recent statistics estimate that breast cancer accounts for approximately 25% of all cancer cases diagnosed in women globally (Meer et al., 2021). Despite significant advances in the treatment of breast cancer, the management of this disease remains a considerable challenge. (Barrios, 2022). Given the critical role of the tumor microenvironment in tumor initiation, progression, and metastasis of breast cancer, researchers have been interested in identifying new prognostic markers that can predict patient outcomes and guide treatment decisions (Cheng et al., 2023; de Visser and Joyce, 2023). Several studies have demonstrated that stromal markers, such as stromal fibroblast density, collagen deposition, and lymphocytic infiltration, can predict patient outcomes in breast cancer (Rudnick and Kuperwasser, 2012). Particularly, the breast tumor microenvironment is composed of various cell types, including fibroblasts, myofibroblasts, endothelial cells, immune cells, and mesenchymal stem cells (MSCs), as well as soluble factors and extracellular matrix components (Giorello et al., 2021). However, the majority of stromal cells are fibroblasts, of which 80% are activated and known as cancer-associated fibroblasts (CAFs) (Sappino et al., 1988). Therefore, CAFs have emerged as a crucial mediator of breast tumor-stroma interactions (Giorello et al., 2021; Sarkar et al., 2023). CAFs are a heterogeneous population of spindle-shaped stromal cells that do not express CD34, CD31, but could exhibit positivity for alpha-smooth muscle actin, Fibroblast-specific protein 1 (FSP) and Fibroblast activation protein α (FAP), among other markers (Kalluri and Zeisberg, 2006; Giorello et al., 2021). It is important to highlight that the phenotypic characteristics of CAFs can vary depending on the fibroblast source and the subtype of breast cancer (Elwakeel and Weigert, 2021).

Regards to their origin, CAFs derive from multiple sources, including resident fibroblasts, bone marrow-mesenchymal stem cells, breast tumor epithelial cells (due to their epithelial-mesenchymal transdifferentiation), endothelial cells (due to their endothelial-mesenchymal transdifferentiation), pericytes, and adipose tissue (Elwakeel and Weigert, 2021; Giorello et al., 2021; Sarkar et al., 2023). It has been observed that bone marrow-MSCs can migrate to the primary breast tumor in the early stages of the disease and contribute to tumor development as part of its microenvironment, either as MSCs or by differentiating into CAFs (Karnoub et al., 2007; Raz et al., 2018). In relation to this, it has been found that 90%–95% of human bone marrow-MSCs express CD105 (endoglin) as well as 50% of CAFs in the tumor stroma of breast cancer patients (BCPs) express CD105 (Pasanen et al., 2016). The CD105 antigen plays a crucial role as a co-receptor for transforming growth factor-beta, which is fundamental in regulating cell proliferation, migration, differentiation, and apoptosis (Muñoz et al., 2021). CD105 also plays an important role in maintaining stemness properties and in the migration of bone marrow-MSCs to the primary breast tumor, mediated by transforming growth factor-beta signaling (Muñoz et al., 2021). Within the tumor microenvironment, transforming growth factor-beta promotes MSC differentiation into CAFs, stimulates their proliferation and activation, and enhances their anti-tumoral activity by inducing the release of extracellular matrix components and other factors (Heneberg, 2016).

As a result of all mentioned above, CAFs have been observed to play an important role as biomarkers in the clinical diagnosis, therapy, and prognosis of this type of cancer (Conklin and Keely, 2012; Ruocco et al., 2018; Salimifard et al., 2020). In previous work, we observed that CD34 (−) stromal cells with a spindle shape, not associated with the vasculature, in invasive ductal breast cancer tissue expressed CD105 but we did not observe its expression in nonmalignant breast tissue (Martinez et al., 2015). Furthermore, our research revealed a significant correlation between high CD105 expression and the development of metastasis in early invasive ductal BCPs (n = 56) (Martinez et al., 2015). Additionally, patients with high CD105 expression experienced shorter metastasis-free survival (MFS) and overall survival rates (OS) (Martinez et al., 2015). However, more efforts are needed to determinate the specific metastatic site.

In the present study, we investigated whether the expression of CD105 in CAFs could constitute an independent prognostic biomarker to predict the occurrence of metastasis in a specific site in early invasive ductal BCPs (n = 342).

## 2 Materials and methods

### 2.1 Patient sample selection

We carried out a retrospective study that involved 342 consecutive patients who had received surgical treatment for breast cancer at Hospital Italiano in Buenos Aires, Argentina. These patients had a confirmed histological diagnosis of early-stage invasive ductal breast carcinoma (stage I/II), as per the International Union Against Cancer TNM classification system (Brierley et al., 2017). A minimum follow-up period of 10 years was ensured after the surgical procedure. The exclusion criteria comprised neoadjuvant therapies, lack of tissue samples, and/or previous development of another primary tumor. Following surgery, all patients received the appropriate treatment, which included a combination of hormonal therapy, and/or radiotherapy, and/or chemotherapy. The treatment plan was determined based on each patient’s clinical and histopathological characteristics, as well as the guidelines recommended by the European Society for Medical Oncology (Pestalozzi et al., 2005; Senkus et al., 2015). This study received approval from the Ethics Committees of the Instituto de Biología y Medicina Experimental (IBYME) and the Hospital Italiano. Informed consent was obtained from patients or their relatives (IBYME approval: CE 050, and Hospital Italiano approval: nº5009). This research was conducted in accordance with the principles outlined in the Helsinki Declaration. To ensure patient privacy, medical records were anonymized using a numerical code.

### 2.2 Clinicopathological characteristics of breast cancer patients

The data concerning clinical information, including bone pain, and metastasis events were collected in collaboration with pathologists from Hospital Italiano. These pathologists had complete access to all patient clinical records, including medical studies, medications, and follow-up data. We recruited patients diagnosed with invasive ductal breast cancer, early stages (I/II), between 2007 and 2013. It is noteworthy that patient follow-up was consistently updated until April 2023. Clinical characteristics of the patients, which are considered classical prognostic markers, were categorized according to the cut-off values specified in the protocols of the Hospital Italiano (Wernicke et al., 2011). These included: a) age (<50 or ≥50 years); b) tumor size (≤2 or >2 cm); c) histological grade based on the Scarff-Bloom-Richardson grading system (Bloom and Richardson, 1957) categorized as differentiated (G1), intermediate (G2), or poor (G3); d) expression of estrogen receptor (ER), progesterone receptor (PR), and HER2/neu status, classified as negative or positive according to Wernicke M et al. (Wernicke et al., 2011) and e) presence of regional metastatic lymph nodes, recorded as negative (no involvement in axillary dissection or sentinel lymph node) or positive (including micro-metastasis). Additionally, the breast cancer subtypes were identified as follows: i) *Luminal A:* This subtype is characterized by positive expression of estrogen receptor (ER) and/or progesterone receptor (PR), a low Ki-67 proliferation index (≤14%), and negative human epidermal growth factor receptor 2 (Her2) expression. ii) *Luminal B:* This subtype expresses ER and/or PR, but usually has a higher Ki-67 proliferation index (>14%) and positive/negative Her2 expression (+). iii) *Basal-like:* Also known as triple-negative breast cancer, this subtype lacks expression of ER, PR, and Her2. iv) *Her2/neu overexpression:* This subtype is ER-negative, PR-negative, and Her2-positive (++++).

Outcome data included local relapse, the occurrence of metastasis, the occurrence of bone metastasis (BM), the occurrence of visceral metastasis (VM), the occurrence of mix (bone and visceral) metastasis (mixM), local relapse-free survival (RFS), MFS, bone metastasis-free survival (BMFS), visceral metastasis-free survival (VMFS), mix metastasis-free survival (mix-MFS), and OS. MFS, BMFS, VMFS, and mix-MFS were defined as the time interval from the date of surgery to the first observation of tumor occurrence (metastatic event and/or local relapse) or last follow-up. Patients included in the mix-metastasis group were those who, at the time of follow-up, exhibited both bone and visceral metastasis. It was not possible to differentiate which event occurred first. The patients with brain (n = 5) and skin (n = 1) metastasis were included in the visceral metastasis group. The methods used for diagnosing metastases included various imaging techniques, such as computed tomography (CT), scintigraphy, and positron emission tomography (PET). Specifically, PET and scintigraphy were used to detect bone metastases, while CT was utilized to identify visceral metastases. The detailed reports obtained from these imaging studies not only revealed the presence of foci consistent with bone and/or visceral metastases but also specified whether these foci were single or multiple within the same organ. In cases where doubts arose regarding the metastasis diagnosis based on the imaging techniques mentioned above, the treating physicians of the patients performed biopsies to confirm the histopathological diagnosis. However, it is important to highlight that the majority of patients with metastasis occurrence in our study did not require biopsies for confirmation.

OS was defined as the interval from the date of surgery until death or last follow-up (Martinez et al., 2015). The specific clinical characteristics of the patients and their corresponding outcome data included in our study were detailed in Table 1. The site of breast cancer metastasis was described in Table 2.

**TABLE 1:** Association of CD105 expression in stromal cells with clinicopathological characteristics (classical prognostic markers), local occurrence, metastatic occurrence, bone metastatic occurrence, visceral metastatic occurrence, and mix metastatic occurrence in 342 patients with early invasive ductal breast cancer. Fisher’s exact test was used for the association between variables, * *p*-value <0.050. of 342 untreated early breast cancer patients. ER *estrogen receptor*, PR *progesterone receptor*.

Clinicopathological characteristics		CD105 expression						
		n	Low expression		High expression		p	
			n	%	n	%		
***Age (years)***	<50	89	51	57.30	38	42.70	0.027*	
	≥50	253	178	70.36	75	29.64		
***Tumor size (cm)***	≤2	218	160	73.39	58	26.61	0.001*	
	>2	124	69	55.65	55	44.35		
***ER***	Negative	44	25	56.82	19	43.18	0.169	
	Positive	298	204	68.46	94	31.54		
***PR***	Negative	59	38	64.41	21	35.59	0.651	
	Positive	283	191	67.49	92	32.51		
***Her2/neu***	Negative	283	194	68.55	89	31.45	0.128	
	Positive	59	34	57.63	25	42.37		
***Histological grade***	G1	35	27	77.14	8	22.86	0.419	
	G2	182	120	65.93	62	34.07		
	G3	125	82	65.60	43	34.40		
***Regional lymph nodes***	Negative	234	163	69.66	71	30.34	0.138	
	Positive	108	66	61.11	42	38.89		
***Local relapse***	Negative	302	201	66.56	101	33.44	0.724	
	Positive	40	28	70.00	12	30.00		
***Metastatic occurrence***	Negative	265	191	72.08	74	27.92	0.0003*	
	Positive	77	38	49.35	39	50.65		
***Bone metastatic occurrence***	Negative	313	226	72.20	87	27.80	0.0005*	
	Positive	29	3	10.34	26	89.66		
***Visceral metastatic occurrence***	Negative	302	199	65.89	103	34.11	0.287	
	Positive	40	30	75.00	10	25.00		
***Mix-metastatic occurrence***	Negative	334	224	67.07	110	32.93	0.722	
	Positive	8	5	62.50	3	37.50		

The values in bold are those that showed significant differences (p < 0.05).

**TABLE 2:** Details of the metastatic sites included in the study.

Metastasis site		Patients (n)	Patients (%)
***Bone metastasis***	Costal arches	6	20.69
	Vertebrae	13	44.83
	Iliac crest and ischium	1	3.45
	Vertebrae-iliac crest-femur	1	3.45
	Femur	1	3.45
	Calota-femur-humerus	1	3.45
	Femur-sternum-vertebrae	2	6.90
	Humerus-iliac crest	1	3.45
	Sternum-vertebrae-pelvis	3	10.34
***Visceral metastasis***	Lung	13	32.50
	Pleura and supraclavicular nodes	4	10.00
	Liver	16	40.00
	Lung-liver-brain	1	2.50
	Brain	5	12.50
	Skin	1	2.50
***Mix metastasis***	Vertebrae-Sternum-Lung	3	37.50
	Vertebrae-Liver-Lung	1	12.50
	Liver-vertebrae	2	25.00
	Lung-vertebrae	1	12.50
	Lung-sternum	1	12.50

The values in bold are those that showed significant differences (p < 0.05).

Furthermore, data regarding the presence of single or multiple foci of metastasis within the same organ were documented. In addition to other clinical data, we included the recording of patients who reported experiencing frequent and persistent bone pain before the onset of metastasis. To document the presence or absence of bone pain, rather than assessing its severity, we did not use any pain assessment form. The patients themselves provided this information in response to their oncologist’s inquiry about its presence, and it was subsequently entered into the patient’s portal by their attending oncologist. It is important to note that patients who experienced bone pain prior to the onset of bone metastases were not undergoing hormonal treatment, chemotherapy, and/or radiotherapy that could bias the origin of bone pain. This precaution was taken to ensure the accuracy and relevance of the data concerning bone pain in our study population.

### 2.3 Tissue processing and analysis of the expression of CD105 in cancer-associated fibroblasts

We worked with paraffin-embedded breast cancer samples fixed in 10% neutral buffered formalin, which were obtained from the surgical archives of the Pathology Department at the Italian Hospital, Buenos Aires, Argentina. The samples were cut into sections with a thickness of 4 μm. The tissue sections were deparaffinized and hydrated through passages in xylene and 100%, 96%, and 70% ethanol. Subsequently, they were incubated in citrate buffer (anhydrous sodium citrate, #7171, Anedra, Buenos Aires, Argentina) at 98 °C for 20 min. To block endogenous peroxidase activity, the tissues were treated with 3% hydrogen peroxide for 5 min. Following that, protein blocking was performed using 1% bovine serum albumin in PBS for 1 h. The tissue sections were then incubated overnight at 4 °C in a humid environment with the anti-CD105 primary human antibody (goat IgG; AF1097; R&D Systems). The LSAB + System-HRP (K0690, Dako, Santa Clara, CA, United States) and 3–3′-diaminobenzidine (Liquid DAB + Substrate Chromogen System; K3468, Dako, Santa Clara, CA, United States) were used according to the manufacturer’s instructions. Subsequently, the tissue sections were incubated overnight with the anti-CD34 antibody (mouse IgG1; M7165; Dako), which was then detected using Biotinylated anti-mouse IgG (H + L; BA-2000; Vector Laboratories), the Vectastain ABC-Alkaline Phosphatase kit (Ak-5000; Vector Laboratories), and the Vector Red Substrate Kit (SK-5100; Vector Laboratories) following the manufacturer’s instructions. Hematoxylin (#121, Biopur, Rosario, Santa Fe, Argentina) was employed for counterstaining, followed by mounting with Canada Balsam (#141, Biopur, Rosario, Santa Fe, Argentina). Negative controls were conducted by incubating tissue sections without primary antibodies, along with irrelevant goat IgG (AB-108-C; R&D Systems) and mouse IgG1 (MAB002; R&D Systems). Duplicate assays were performed for each sample.

Cells with membranous staining, nuclear counterstaining, and displaying characteristic fibroblastic morphology (spindle shape) were counted within the intratumoral stroma. Enumeration was performed in five representative optical field areas per tissue section at a magnification of ×400. To evaluate the CD105 immunohistochemical signal, a quantitative score was assigned based on the percentage of stromal cells exhibiting positive staining [0% (score 0); <10% (score 1); 10%–50% (score 2); 51%–80% (score 3); or >80% (score 4*)*] as we previously determined (Martinez et al., 2015). Additionally, the intensity of staining in positive cells was scored [absent (score 0); low (score 1); moderate (score 2); or intense (score 3) compared to an internal control]. The quantitative score and intensity score were added to get a total score ranging from 0 to 7. The CD105 intensity score was determined by comparing the staining intensity of stromal cells with that observed in endothelial cells of breast tissue (Charpin et al., 2004; Dales et al., 2004; Li et al., 2011; Rau et al., 2012). The assessment of slides was conducted independently by two pathologists. There was an agreement of 87.5% in the immunohistochemical evaluation between the two observers (Kappa value = 0.840).

### 2.4 Analysis of intratumoral stromal characteristics

The study of tumor stromal histological features as intratumor stroma, amount of fibroblasts, collagen deposition, lymphocytic infiltration, myxoid changes (Wernicke et al., 2003), blood and lymphatic vascularization, and presence of anarchic microcalcifications were determined by hematoxylin and eosin staining (de Kruijf et al., 2011). Specifically, the intratumor stroma was quantified as a percentage, categorized as the low amount (<50%) or high amount (≥50%). In addition, pathologists scored the presence of fibroblasts, collagen deposition, lymphocytic infiltration, myxoid changes, blood and lymphatic vascularization, and microcalcifications using a scale of absent (0%, score 0), scanty (<30%, score 1), moderate (30%–50%, score 2), or abundant (≥50%, score 3).

The analysis of anarchic microcalcifications in each breast cancer paraffin block was cross-referenced with the data from the patient’s mammograms to confirm their presence. Another piece of information collected from the patient’s medical records was the degree of desmoplasia scored as low/moderate (<50%) or severe (≥50%).

### 2.5 Analysis METABRIC dataset

To validate our findings using an independent data cohort, CD105 expression in primary tumors of BCPs was obtained from the METABRIC cohort, which is available in the cBioPortal bioinformatics tool (http://www.cbioportal.org) (Cerami et al., 2012). The analysis included RNAseq (IlluminaHiSeq) data from 105 BCPs samples (invasive ductal breast carcinoma, clinical stage I/II). The data were filtered based on the expression of the CD34 marker, selecting only the samples with CD34 mRNA expression z-scores ≤0 log2 (RPM+1). In addition, samples were selected with FSP mRNA expression z-scores ≥0 log2 (RPM+1) and FAP mRNA expression z-scores ≥0 log2 (RPM+1) to ensure that we are working with cancer-associated fibroblasts. Subsequently, the CD105 expression was scored based on the median (−0.29 log2 [RPM+1]), resulting in two data populations corresponding to low and high CD105 expression (n = 51 and n = 54, respectively).

### 2.6 Statistical analysis

To assess the relationship between CD105 expression in spindle-shaped stromal cells, not associated to the vasculature (CAFs) with other intratumoral stromal characteristics, as well as with clinicopathological features in breast cancer patients (BCPs), we employed a cut-off value-based approach, which is a common technique in biomarker and quantitative characteristic analysis (Martinez et al., 2015). In the first step, we determined the cut-off values for each of the studied parameters, including CD105 expression and other tumor-related factors, based on the values of the first quartile (Q1), the median (M), and the third quartile (Q3). Subsequently, univariate analyses were conducted using these cut-off values to examine their association with OS in BCPs. The univariate analysis allowed us to assess the relationship between each parameter and OS individually, without considering other variables. The cut-off value with the lowest *p*-value in this univariate analysis was considered the optimal cut-off for that specific characteristic. A lower *p*-value indicates a stronger association between the characteristic and OS, suggesting that the observed relationship is less likely to occur by chance. The optimal cut-off values for the expression of CD105, as well as for the amount of fibroblasts, collagen deposition, lymphocytic infiltration, myxoid changes, blood and lymphatic vascularization, were as follows: 3 (M), 1 (Q1), 2 (Q3), 0 (Q1), 2 (Q3), 1 (Q1), and 0 (Q1), respectively. In this way, the CD105 expression, percentage of fibroblasts, collagen deposition, lymphocytic infiltration, myxoid changes, blood and lymphatic vascularization, and presence of microcalcifications were categorized as either absent/scanty or large amounts according to the selected optimal cut-off value.

We used Fisher’s exact test to evaluate the association of fibroblast CD105 with classical prognostic markers, as well as local relapse, metastatic occurrence, bone metastatic occurrence, visceral metastatic occurrence, and mixed metastatic occurrence.

Survival analyses including RFS, MFS, BMFS, VMFS, mix-MFS, and OS were conducted using the Kaplan-Meier method, and the log-rank (Mantel-Cox) test (Senkus et al., 2015) was used to assess differences. Multivariate survival analysis was performed using the Cox proportional hazards model with backward stepwise selection (likelihood ratio), considering only the significant variables identified in the univariate analysis. A significance level of 0.05 was set for all analyses. Statistical analysis was carried out by an experienced statistician using SPSS software (version 18.00, Chicago, Illinois).

## 3 Results

**FIGURE 1:**
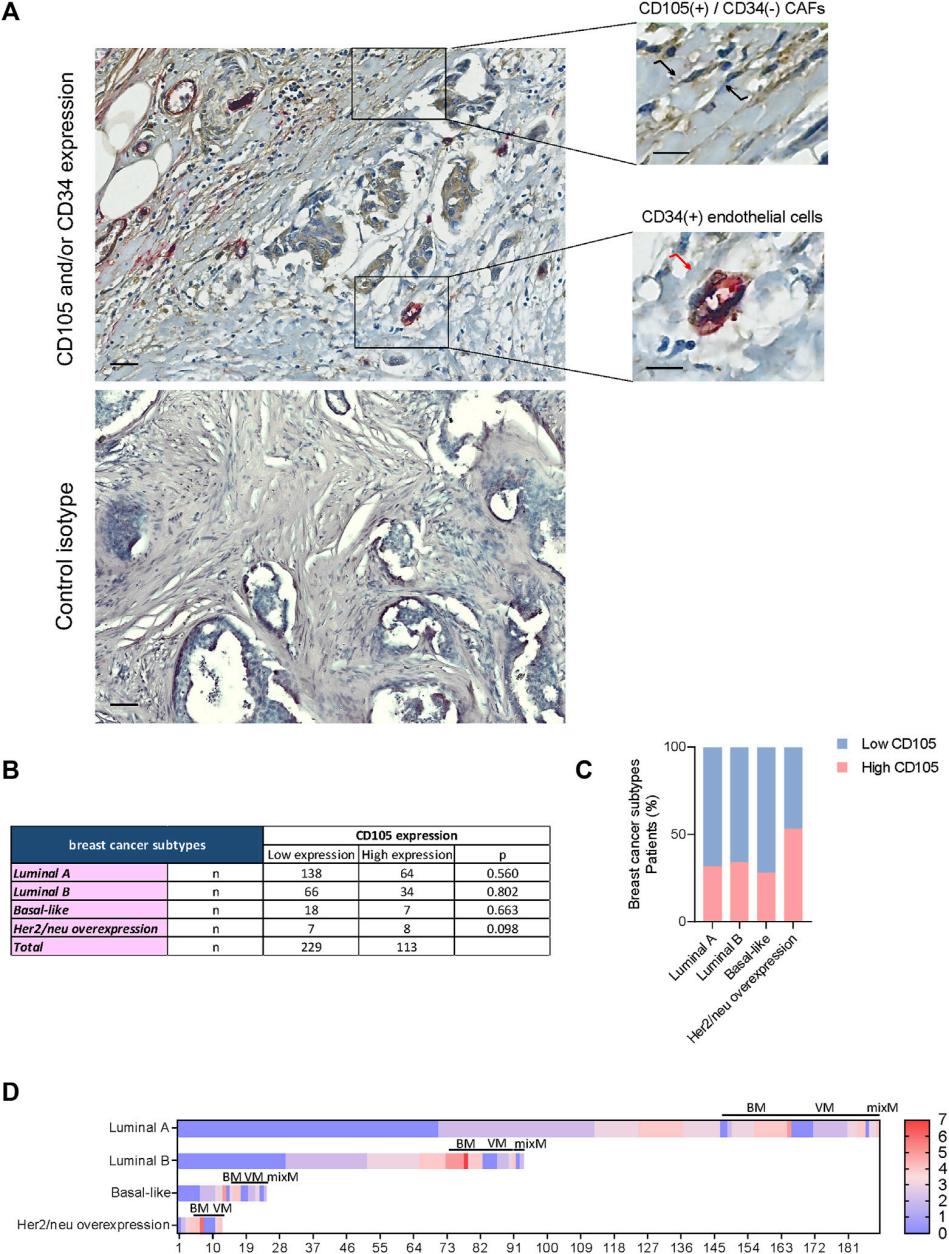
**(A)** Expression of CD105 and CD34 in stromal cells from the primary tumor of breast cancer patients. *Top panel:* Double immunohistochemistry for CD105 and CD34 (detected by brown and red chromogen, respectively) presents a representative example of co-staining of CD105 and CD34 in endothelial cells (•) and exclusive CD105-positive staining in evaluated stromal cells (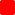) of primary tumor tissue from a breast cancer patient. *Bottom panel:* Negative isotype controls. Nuclei were counterstained with hematoxylin (purple). Original magnification: ×200. Scale bars represent 200 μm and 100 μm in the *inset*. **(B)** Expression of CD105 in breast cancer and its different subtypes. Association of CD105 expression in cancer-associated fibroblast with different breast cancer subtypes. Fisher’s exact test was used to assess the association between variables, * *p*-value <0.050. **(C)** Histogram representing the association of CD105 expression in these stromal cells with different breast cancer subtypes. **(D)** Heatmap illustrating the distribution of CD105 expression across all samples included in the study, including cases with bone, visceral, and mix metastasis (BM, VS. mix M, respectively).

In the initial stage, we performed a double CD105-CD34 immunohistochemical analysis to ensure that the readings were specifically obtained from spindle-shaped stromal cells not associated with the vasculature (CD34 negative) (CAFs). This approach allowed us to discard the possibility of false positives by mistakenly identifying endothelial progenitors. The analysis demonstrated that the spindle-shaped stromal cells, not associated with the vasculature, were positive for CD105 and displayed a lack of CD34 staining (Figure 1A).

### 3.1 CD105 expression in cancer-associated fibroblasts

Out of a total of 342 BCPs diagnosed with invasive ductal breast cancer (stage I/II), 113 (33.04%) samples were found to have high CD105 expression (Figure 1B). The expression of CD105 was not significantly associated with a specific subtype of breast cancer (Figures 1B,C). The distribution of CD105 expression across different breast cancer subtypes was displayed as a heat map (Figure 1D), including BCPs with and without metastatic occurrence (bone, visceral, and mix).

### 3.2 Association between CD105 expression in cancer-associated fibroblasts, and clinicopathological characteristics of breast cancer patients

It was found that CD105 expression was significantly associated with patient age (*p* = 0.027, Table 1). The 42.70% of patients with age <50 years had a high CD105 expression, while the 29.64% of patients with age ≥50 years had a high CD105 expression (Table 1).

Furthermore, it was found that CD105 expression was significantly associated with tumor size in our BCPs (*p* = 0.001, Table 1). The 44.35% of BCPs with tumor size >2 cm had high CD105 expression, while 26.61% of BCPs with tumor size ≤2 cm also had high expression of this marker (Table 1).

No significant differences were found regarding the association of CD105 expression

with other classical parameters such as ER, PR, and Her2/neu status, histological grade, and regional lymph node status (Table 1).

High CD105 expression was significantly associated with a greater risk of developing metastasis (*p* = 0.0003, Table 1). BCPs with high CD105 expression exhibited a metastatic occurrence rate of 34.51%, whereas the group with low CD105 expression had only 16.59% experiencing the metastatic event. It can also be observed that among patients who experienced metastasis, 50.65% had high CD105 expression (Table 1). When analyzing CD105 expression considering the site of metastasis, we found that 89.66% of BCPs with BM had high CD105 expression (*p* = 0.0005, Table 1). Within the group of BCPs with high CD105 expression, 23.01% had BM, while within the group of BCPs with low CD105 expression, only 1.31% had this event.

No significant association was found between CD105 expression and other sites of metastasis, such as visceral and/or mix (simultaneous visceral and bone), as well as between CD105 expression and local relapse (Table 1).

The expression of CD105 was also associated with the number of metastatic foci per organ, both overall and in bone (*p* = 0.001 and *p* = 1.19 × 10^−11^, respectively) (Table 3). It was found that patients with high CD105 expression exhibited multiple foci within the same organ (Table 3). This same pattern was also observed when analyzing bone metastasis (Table 3).

**TABLE 3:** Association of CD105 expression in stromal cells with a number (#) of metastatic foci per organ overall and (#) of metastatic foci per bone organ in 342 patients with early invasive ductal breast cancer. Fisher’s exact test was used for the association between variables, * *p*-value <0.050.

*Characteristics of the metastatic focus*	*n*	*# metastatic foci*		*# Bone metastatic foci*	
		*>1 focus (n)*	*p*	*>1 focus (n)*	*p*
*CD105*					
Low expression	229	20	0.001*	2	**1.19 × 10** ^**−11**^ *
High expression	113	25		19	

The values in bold are those that showed significant differences (p < 0.05).

**FIGURE 2:**
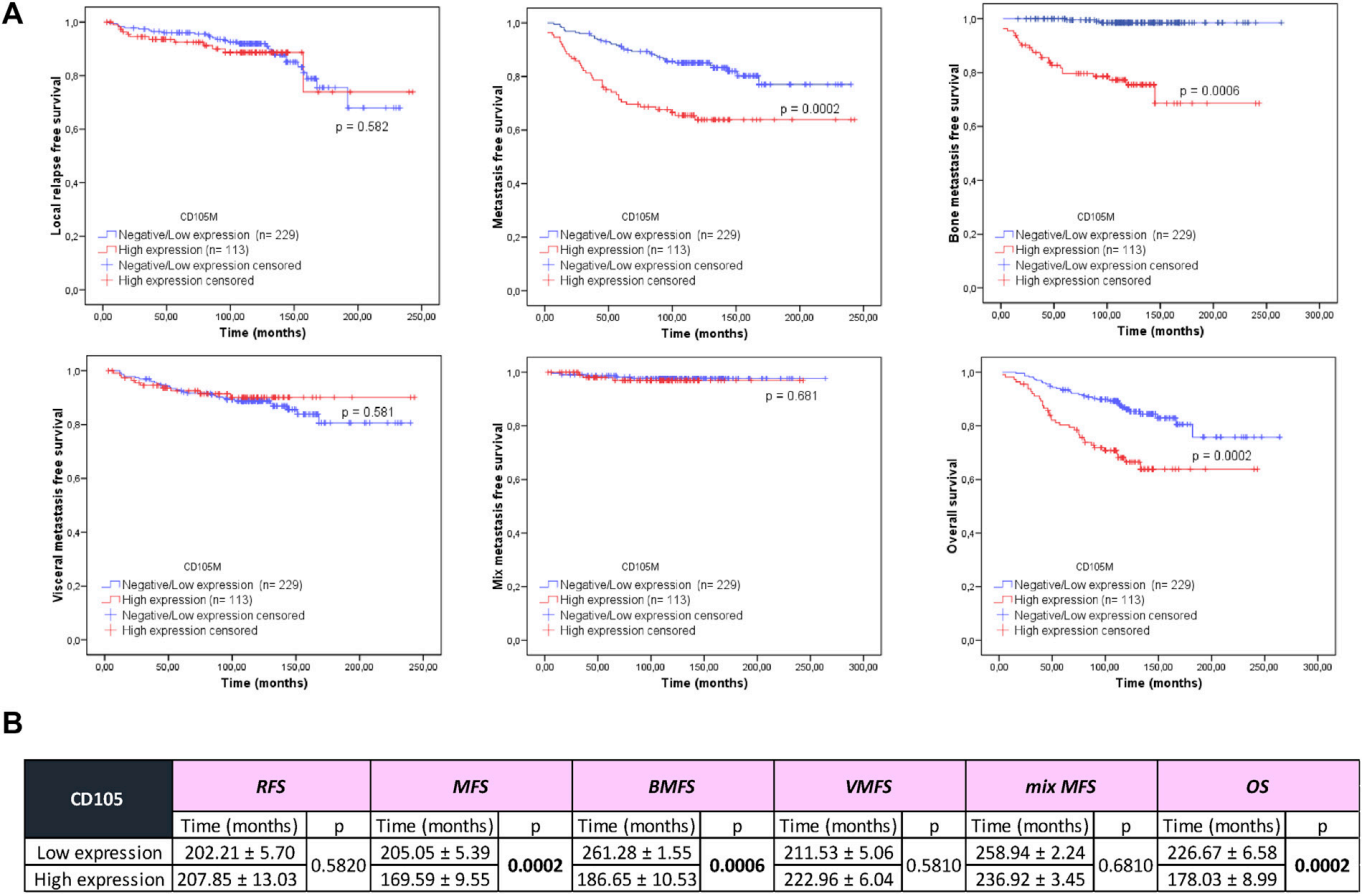
Association of CD105 expression with local relapse-free survival (RFS), metastasis-free survival (MFS), bone metastasis-free survival (BMFS), visceral metastasis-free survival (VMFS), mix metastasis-free survival (mix MFS), and overall survival (OS) in patients with early invasive ductal breast cancer. **(A)** Kaplan-Meier curves (univariate analysis) marked in red represent data from samples with high CD105 expression, while blue curves represent samples with negative/low CD105 expression. Log Rank (Mantel-Cox) test was used to assess the Kaplan-Meier curves. * *p*-value < 0.050. **(B)** Details of local RFS, MFS, BMFS, VMFS, mix MFS, and OS correspond to the low and high CD105 expression groups.

CD105 expression was significantly associated with MFS, BMFS, and OS (*p* = 0.0002, *p* = 0.0006, and *p* = 0.0002, respectively) (Figure 2A). BCPs with high CD105 expression had shorter MFS (169.59 ± 9.55 months), shorter BMFS (186.65 ± 10.53 months), and lower OS (178.03 ± 8.99 months) compared to the group of patients with low CD105 expression (205.05 ± 5.39, 261.28 ± 1.55, and 226.67 ± 6.58 months, respectively) (Figure 2B).

### 3.3 CD105 expression in cancer-associated fibroblasts and its relationship with anarchic microcalcifications and bone pain

**FIGURE 3:**
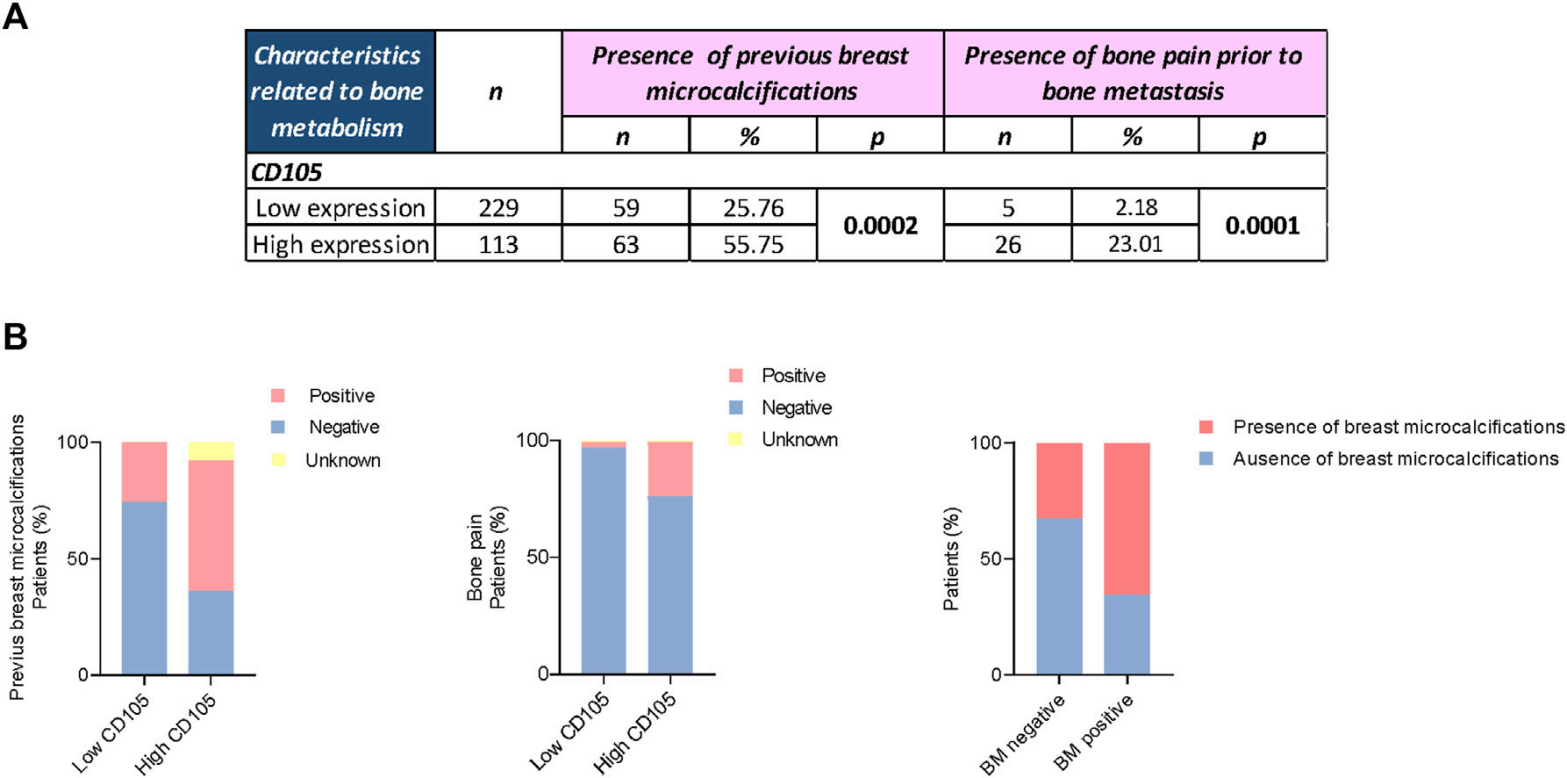
CD105 expression in CAFs and its relationship with anarchic breast microcalcifications and bone pain. **(A)** Association of CD105 expression with the presence of anarchic microcalcifications and bone pain prior to bone metastasis. Fisher’s exact test was used to assess the association between variables, * *p*-value <0.050. **(B)** Graphical representation of the aforementioned associations and the presence of microcalcifications and bone metastasis (BM).

**FIGURE 4:**
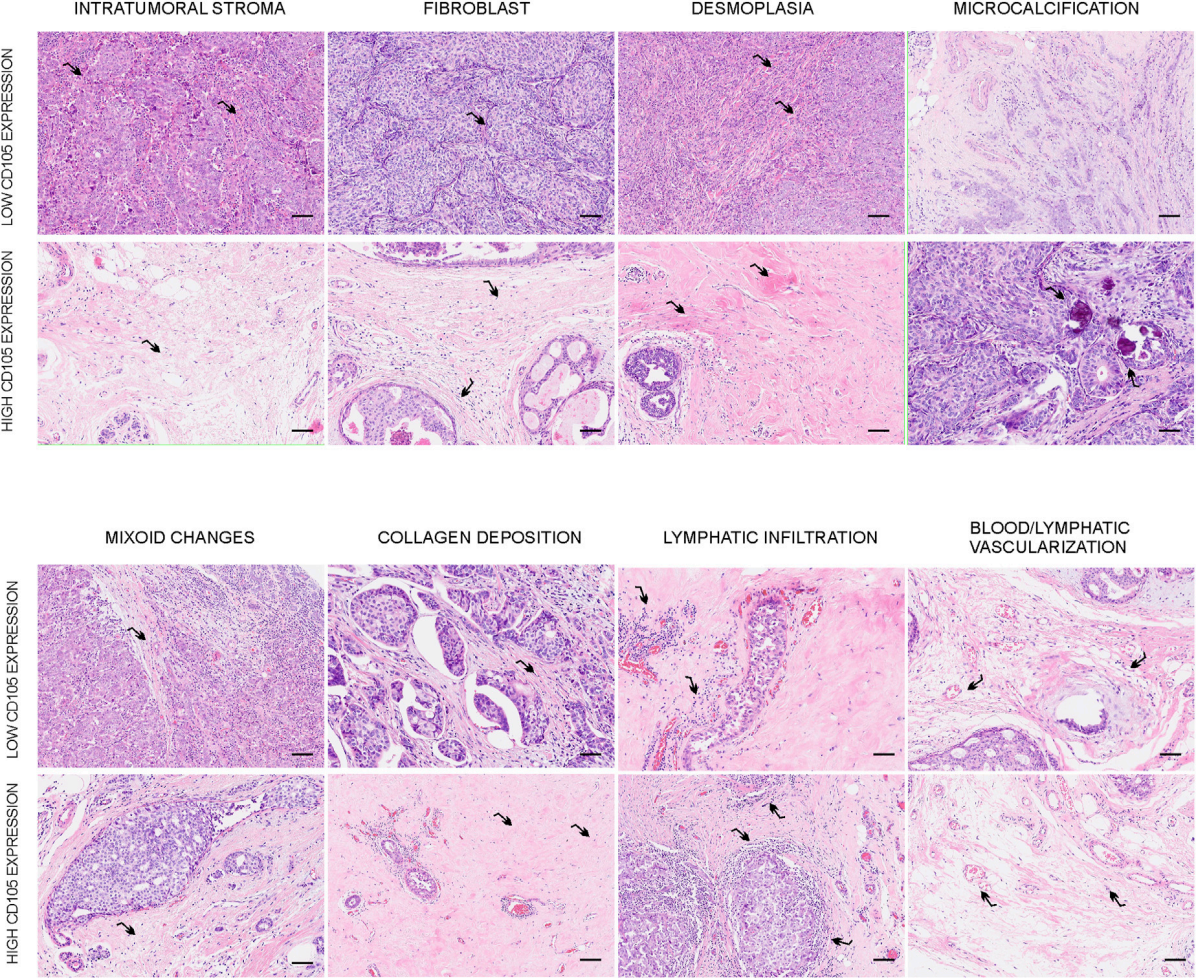
Representative images of the relationship between histological characteristics of the breast tumor and CD105 expression. Hematoxylin and eosin staining were utilized to assess the histological characteristics of the stroma in the primary breast tumor. This figure illustrates samples exhibiting different levels of intratumoral stroma, fibroblasts, desmoplasia, myxoid changes, microcalcifications, collagen deposition, lymphocytic infiltration, and blood/lymphatic vascularization in relation to the expression of CD105. The images were captured at an original magnification of ×200, and the scale bars represent 200 μm.

CD105 expression was found to be associated with the presence of anarchic microcalcifications in the primary breast tumor (*p* = 0.0002) (Figures 3A,B; Figure 4). Among BCPs with high CD105 expression, 55.75% exhibited anarchic microcalcifications, compared to 25.76% of BCPs with low CD105 expression (Figures 3A,B). In addition, we found a significant association between the presence of microcalcifications and the occurrence of BM. Among the patients in our cohort who developed BM (29 out of a total of 342 patients), 65.52% exhibited the presence of anarchic microcalcifications in the breast prior to breast surgery (Figure 3B,). Also, a higher proportion of patients with high CD105 expression experienced bone pain prior to the onset of metastatic events compared to the group of BCPs with low CD105 expression (*p* = 0.0001, 23.01% vs. 2.18%) (Figures 3A,B).

### 3.4 Association between CD105 expression in cancer-associated fibroblasts and intratumoral stroma characteristics

CD105 expression was associated with the percentage of intratumoral stroma, the percentage of fibroblasts, and desmoplasia (*p* = 7.05 × 10^−8^, *p* = 0.004, and *p* = 0.028, respectively, Table 4). Of the patients with high CD105 expression, 63.71% exhibited a high percentage of intratumoral stroma, while only 32.75% of patients with low expression showed a high intratumoral stroma (Table 4; Figure 4). Regarding the abundance of fibroblasts, high CD105 expression was associated with a higher quantity of intratumoral fibroblasts. 61.06% of patients with high CD105 expression exhibited a higher number of fibroblasts, whereas only 49.28% of patients with low CD105 expression showed high fibroblast abundance (Table 4; Figure 4). Additionally, CD105 expression was related to the degree of desmoplasia, with 53.09% of patients with high CD105 expression exhibiting severe desmoplasia compared to 37.11% in the low CD105 expression group (Table 4; Figure 4).

**TABLE 4:** Association between stromal histological features and CD105 expression in a cohort of 342 untreated early breast cancer patients. Fisher’s exact test was used for the association between variables, * *p*-value <0.050.

Characteristics of the breast tumor stroma		CD105 expression	
		Low expression	High expression
***% Intratumoral Stroma***	***> 50***	75	72
	***p***	**7.05 × 10** ^**−8**^ *	
***% Fibroblast***	***Large amount***	103	69
	***p***	0.004*	
***Collagen deposition***	***Large amount***	64	36
	***p***	0.452	
***Lymphatic infiltration***	***Large amount***	108	56
	***p***	0.730	
***Desmoplasia***	***Large amount***	85	60
	***p***	0.028*	
***Mixoid changes***	***Large amount***	15	10
	***p***	0.510	
***Blood vascularization***	***Large amount***	57	35
	***p***	0.172	
***Lymphatic vascularization***	***Large amount***	85	50
	***p***	0.075	
**Total**		229	113

The values in bold are those that showed significant differences (p < 0.05).

The association between stromal histological features and classical prognostic markers, as well as local relapse, metastatic occurrence, bone metastatic occurrence, visceral metastatic occurrence, and mix metastatic occurrence, is shown in Supplementary Table S1.

### 3.5 Association between classical prognostic markers and tumor progression

Univariate survival analysis showed that ER status, PR status, tumor size, histological grade, and presence of micro/macrometastasis in regional lymph nodes were significantly associated with worse prognosis of BCPs in our cohort. Patients with ER (−) or PR (−) had lower MFS, BMFS, VMFS, and OS (*p* = 0.0007, *p* = 0.003, *p* = 0.001, *p* = 0.0005, respectively; and *p* = 0.0002, *p* = 0.019, *p* = 0.011, *p* = 0.0003, respectively). Furthermore, patients with tumor size>2 cm had lower MFS, BMFS, VMFS, MMFS, and OS (*p* = 0.0007, *p* = 0.001, *p* = 0.0002, and *p* = 0.0003, respectively). In addition, BCPs with high differentiation grade (G3) had lower MFS, BMFS, and OS (*p* = 0.0004, *p* = 0.011, and *p* = 0.003, respectively). Finally, patients with the presence of micro/macrometastasis in regional lymph nodes had lower MFS (*p* = 0.046) (Data not shown).

### 3.6 Multivariate analysis

The multivariate analysis revealed that CD105 expression was an independent predictor for MFS, BMFS, and OS in our BCPs (*p* = 0.0003, *p* = 0.0006, and 0.0001, respectively) (Table 5). Unfortunately, lymph nodes status was not an independent prognostic factor for MFS, like other authors found (Weissenbacher et al., 2010; Bitencourt et al., 2020; Olfatbakhsh et al., 2022). This result may be attributed to the selection of early primary breast tumors and/or the small cohort size.

**TABLE 5:** Multivariate analysis of metastasis-free survival, bone metastasis-free survival overall survival in 342 patients with early invasive ductal breast cancer. C.I.: Confidence Interval; HR: Hazard Ratio. Statistical test: Cox proportional hazards model (backward stepwise selection), * *p*-value <0.050. ER *estrogen receptor*, PR *progesterone receptor*.

Events	Characteristics	RR	95% C.I.	*p*
***Metastasis-free survival***	ER	0.459	0.211–1.001	0.050*
	PR	0.836	0.381–1.835	0.656
	Tumor size	2.869	1.738–4.736	0.0003*
	Histological Grade	1.339	0.853–2.103	0.205
	**CD105**	**2.299**	**1.452–3.639**	0.0003*
	Micro/macrometastasis in regional lymph nodes	1.191	0.727–1.950	0.487
***Bone metastasis-free survival***	ER	0.452	0.192–1.067	0.07
	Tumor size	1.842	0.835–4.064	0.13
	Histological Grade	2.233	1.071–4.658	0.032*
	**CD105**	**21.234**	**6.357–70.928**	00,006*
***Overall survival***	ER	0.4890	0.217–1.104	0.085
	PR	2.8910	1.718–4.867	0.0006*
	Tumor size	0.6150	0.276–1.415	0.260
	Histological Grade	1.2050	0.750–1.935	0.441
	**CD105**	**2.5380**	**1.566–4.114**	0.0001*

The values in bold are those that showed significant differences (p < 0.05).

### 3.7 Prognostic relevance of CD105 mRNA expression in FSP (+), FAP (+), and CD34 (−) cells within the breast cancer tumor using a bioinformatics approach

**FIGURE 5:**
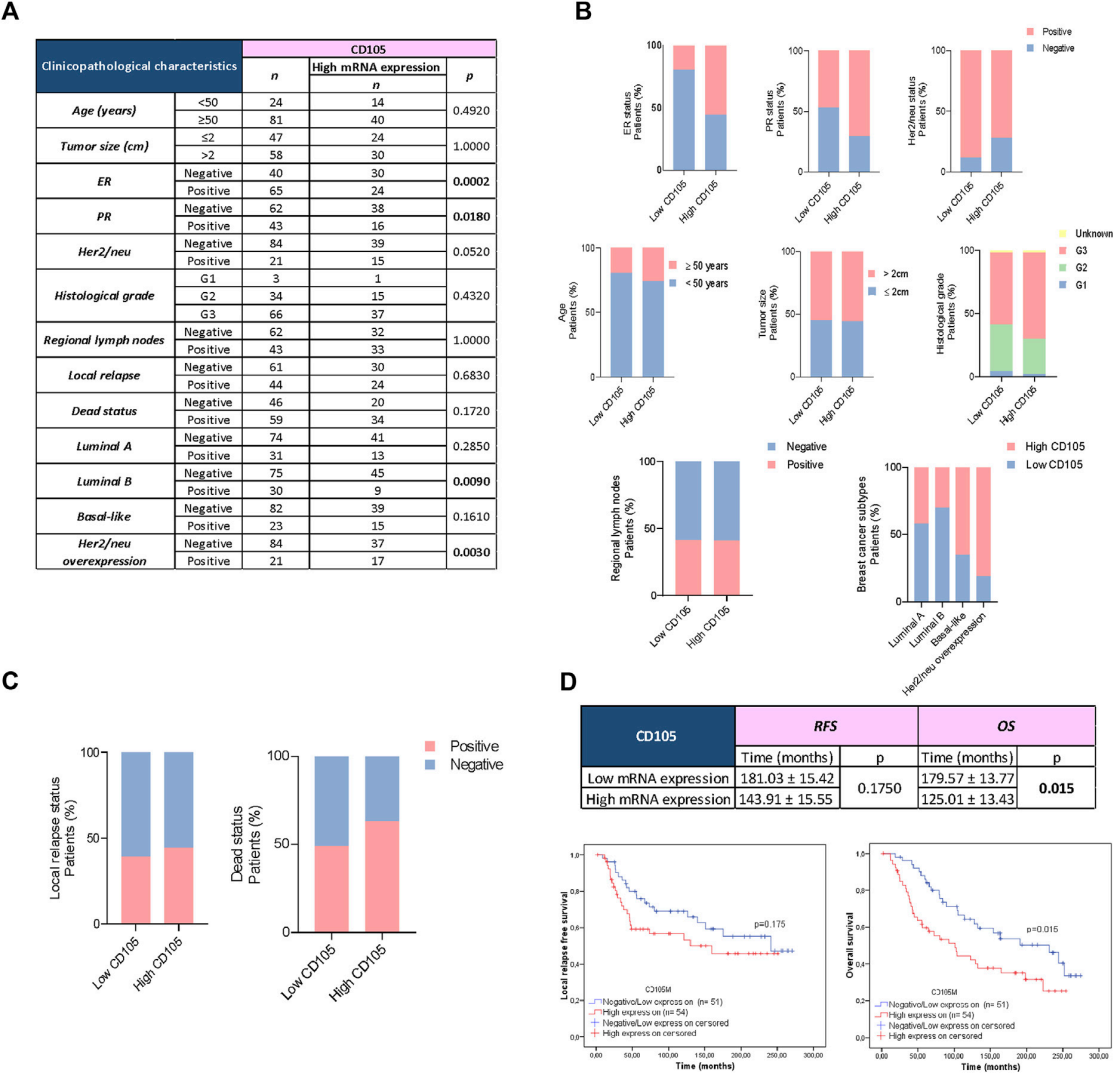
CD105 mRNA Expression in FSP (+), FAP (+), and CD34 (−) cells within the breast cancer tumor using a bioinformatics approach. **(A)** Association of CD105 expression in stromal cells (fibroblast-like) with classical prognostic markers, local relapse, metastatic occurrence, bone metastatic occurrence, visceral metastatic occurrence, and mix metastatic occurrence in 105 patients with early invasive ductal breast cancer from the METABRIC breast cancer dataset. Fisher’s exact test was utilized to assess the association between variables, with a * *p*-value <0.050. **(B)**, **(C)** Graphical representation of the associations between CD105 expression and classical prognostic factors, as well as other clinicopathological data. **(D)** Kaplan-Meier curves (univariate analysis) marked in red represent data from samples with high CD105 expression, while blue curves represent samples with negative/low CD105 expression. The Log Rank (Mantel-Cox) test was employed to evaluate the Kaplan-Meier curves, with a * *p*-value <0.050.

In order to validate our results in another data cohort, we used the METABRIC breast cancer cohort database available in the cbioportal bioinformatics tool. We found that the mRNA expression of CD105 was associated with the status of ER and PR. Among patients with negative ER expression, 75% showed high expression of CD105 mRNA (*p* = 0.0002) (Figures 5A,B). Furthermore, when analyzing the population of patients with negative PR, we found that 61.29% had elevated expression of CD105 (*p* = 0.0180) (Figures 5A,B). In relation to this result, we observed that the group of patients with high expression of CD105 mRNA had a lower proportion of luminal B subtype breast cancer compared to the group with low expression (29.63% vs. 52.94%, respectively, *p* = 0.009) (Figures 5A,B). Additionally, an increased proportion of breast cancer with Her2/neu overexpression was found in the group with high expression of CD105 compared to the group with low expression of this marker (27.78% vs. 11.76%, respectively, *p* = 0.003) (Figures 5A,B). We did not find any significant association between CD105 mRNA expression and patient age, tumor size, histological grade, or the presence of micro/macrometastasis in regional lymph nodes. Finally, we found that CD105 mRNA expression was associated with patient OS (Figures 5C,D). The group of patients with high CD105 expression had lower OS than the group with low expression (*p* = 0.0150) (Figures 5C,D).

## 4 Discussion

Currently, it is known that CAFs are one of the cell populations that strongly promote the progression of breast cancer (Sarkar et al., 2023). However, further research is needed to fully understand their precise role within the breast tumor microenvironment. Determining the specific types of stromal cells and their roles in the tumor environment are crucial for gaining a better understanding of the evolutionary process of breast cancer. The presence of CD105 (+) CAFs within the stroma of breast tumors could potentially indicate the existence of fibroblasts derived from MSCs that may have migrated from the bone marrow during the initial stages of primary tumor development (Karnoub et al., 2007; Raz et al., 2018). In relation with this last comment, in previous studies conducted in our laboratory, we have observed that tumor cells of BCPs (invasive ductal carcinoma, stage I/II) produce bone marrow-MSCs chemotactic substances, such as IL-6, SDF-1, and CCL-2, among others. Additionally, we have found a significant association between the expression of these ligands in breast tumor cells and the expression of their respective receptors, IL-6R, CXCR-4, and CCR-2, in spindle-shaped stromal cells, not associated to the vasculature, found within the tumor microenvironment of these BCPs (Labovsky et al., 2015).

When dividing our cohort of BCPs based on CD105 expression, we found that CD105 expression was associated with age and tumor size. We observed that patients who were younger than 50 years old, as well as those who had a tumor size greater than 2 cm, had a higher abundance of CD105 (+) CAFs. It is well known that younger patients or those with larger tumor sizes tend to have a more aggressive tumor profile and worse prognosis in breast cancer (Rosen et al., 1981; Ma et al., 2020). Hence, the presence of spindle-shaped stromal cells lacking CD34 expression, and displaying CD105 expression, could potentially indicate an increased tumor aggressiveness in younger patients. This suggests the existence of a tumor microenvironment that facilitates breast tumor progression and growth.

The abundance of stroma, particularly fibroblasts, and the presence of desmoplasia in the tumor microenvironment of breast cancer have emerged as crucial factors influencing tumor progression and patient outcomes (Walker, 2001; de Kruijf et al., 2011; Cid et al., 2017; Yamauchi et al., 2018; Saini et al., 2020). Fibroblasts within the stroma play multifaceted roles in promoting tumor growth, angiogenesis, and metastasis, while desmoplasia reflects the deposition of extracellular matrix components, particularly collagen fibers, contributing to a fibrotic microenvironment (Zeltz et al., 2020). These components of the tumor stroma create a supportive niche for tumor cells, facilitating their survival, invasion, and dissemination (Sarkar et al., 2023). The results of our study revealed a significant association between CD105 expression and the percentage of intratumoral stroma, the abundance of fibroblasts, and the degree of desmoplasia. Those BCPs with a high proportion of intratumoral stroma, a high abundance of fibroblasts, or severe desmoplasia had a higher proportion of CD105 (+) CAFs indicating a potential role of CD105-positive fibroblasts in driving the deposition of extracellular matrix components and the development of a fibrotic tumor microenvironment.

The results of this study reveal a significant association between high CD105 expression and an increased risk of metastasis in BCPs. Patients with high CD105 expression showed a higher occurrence rate of metastasis compared to those with low CD105 expression. These findings are consistent with a previous study that has highlighted the role of CD105 in promoting tumor progression and metastatic dissemination (Martinez et al., 2015). Bone represents the predominant site of metastatic spread in BCPs and represents a major contributor to patient mortality (Othman et al., 2021). In relation, Salvador F. et al. described that “…Most cells that escape primary tumors are unable to establish metastatic lesions, which suggests that target organ microenvironments are hostile for tumor cells. This implies that breast cancer cells must achieve a process of speciation to adapt to the new conditions imposed in the new organ. Bone has unique characteristics that can be exploited by cancer cells: it undergoes constant remodeling and comprises diverse environments … ” (Salvador et al., 2019). Interestingly, when considering the site of metastasis, we observed a significant association between CD105 expression and BM. The majority of BCPs with BM exhibited high CD105 expression, suggesting a preferential role of CD105 (+) CAFs in BM occurrence. It suggests that CD105 (+) CAFs could have a significant impact on the development and progression of the primary breast tumor and potentially contribute to the evolution of metastatic cascade, specifically in the bone marrow/bone. In previous work, we found a significant positive association between RANKL and CCL-2 expression in spindle-shaped stromal cells, not associated with the vasculature, in primary tumors of early BCPs, with the receptor expression of RANK, and CCR-2 in breast tumor cells, respectively (Labovsky et al., 2015). These findings, along with the discoveries made by other researchers, suggest that these stromal cells, through the actions of RANKL and CCL-2, have the potential to influence the proliferation, survival, invasion, migration, and intravasation of breast tumor cells during the early stages of this type of cancer (Dwyer et al., 2007; Dittmer et al., 2011; Palafox et al., 2012; Potter et al., 2012; Mimeault and Batra, 2014). However, further studies are warranted to elucidate the underlying mechanisms by which CD105 (+) CAFs, contribute to BM.

We found that the expression of CD105 in CAFs showed significant associations with the number of metastatic foci per organ, particularly in bone. Patients with high CD105 expression had a higher incidence of multiple foci within the same organ compared to those with low CD105 expression, and this trend was particularly evident in BM. This suggests that high expression of CD105 in CAFs may contribute to the establishment and growth of multiple metastatic lesions in specific organ sites, particularly in the bone microenvironment. Furthermore, BCPs with high CD105 expression exhibited shorter MFS, BMFS, and OS. These results emphasize the prognostic value of CD105 as a potential biomarker for BM and survival in BCPs.

Another notable finding was the correlation between CD105 expression and the presence of anarchic microcalcifications in the primary breast tumor. Our results demonstrated that BCPs with high CD105 expression had a significantly higher incidence of anarchic microcalcifications. This suggests that CD105 expression in CAFs may play a role in the development or promotion of microcalcification formation in the breast. Furthermore, we found a significant association between the presence of microcalcifications and the occurrence of BM. The presence of microcalcifications in breast tissue has long been recognized as an important diagnostic feature in breast cancer (Clemenceau et al., 2020). These microcalcifications are often detected through mammography and are associated with various pathological conditions, including both benign and malignant breast lesions (Clemenceau et al., 2020). While the exact mechanisms underlying their formation are not fully understood, emerging evidence suggests that calcification processes in the breast microenvironment involve the presence of cells with osteoblast-like characteristics (Scimeca et al., 2020). Studies revealed that BCPs with microcalcifications exhibited elevated levels of various proteins, like RANKL, SDF-1 and OPN, in comparison to breast cancer cases without microcalcifications (Tan et al., 2016; Scimeca et al., 2020). These findings align with our previous study, which demonstrated the positive expression of the RANKL and SDF-1 markers by tumor cells in early-stage invasive ductal breast cancer (Labovsky et al., 2015). Therefore, we might think that CD105 (+) CAFs may favor the presence of breast tumor cells with characteristics of osteoblasts thus inducing the calcifications formation.

It is known that studying the expression of CD105 in CAFs at the protein level through immunohistochemistry confers us the advantage of directly visualizing the presence and distribution of CD105 in the tumor microenvironment. This technique allows us to assess the localization and abundance of CD105 in the stromal compartment, providing valuable information about its potential role in tumor progression. However, considering that mRNA levels can provide valuable insights into gene expression patterns, we found it intriguing to explore whether the expression of CD105 at the mRNA level also correlates with the patient’s outcome. This additional investigation aims to validate our findings at another level of gene expression, providing a more comprehensive understanding of the potential role of CD105 in the context of the disease. To investigate this, we segregated the data population based on the expression of CD105, FAP and FSP mRNA and the absence of CD34 mRNA expression, considering that the data from these databases includes the entire tumor. In this regard, we obtained similar results to those obtained in our cohort, showing that samples with elevated mRNA expression of CD105 had a shorter OS. However, it is unfortunate that this database does not provide information about the occurrence of metastases in different sites, and the time to these events. Furthermore, we found differences in the association with classical parameters. In our cohort, CD105 expression was associated with tumor size and patient age, which was not found in the METABRIC data cohort. However, in this last data cohort, we did find an association between ER and PR status and CD105 mRNA expression. These differences in the results may be attributed to the various factors, including cohort bias, differences in patient demographics, and overall experimental methodologies.

Therefore, CD105 expression provides a promising parameter that is not only easily detectable but also reproducible and time-efficient across various subtypes of breast cancer. Considering these advantages, CD105 expression in CAFs is a candidate marker that can be easily incorporated into routine pathology diagnostics. Its inclusion would enhance the accuracy of BM risk stratification for BCPs, allowing for better-informed treatment decisions and improved patient outcomes. Further research and validation studies are warranted to establish the clinical utility and long-term benefits of CD105 expression in CAFs as a diagnostic tool in early breast cancer management.

## 5 Conclusion

Our study provides evidence that the presence of CD105 (+) CAFs has a significant impact on the prognosis of BCPs. We found that high CD105 expression in these cells is an unfavorable prognostic marker for women with early invasive ductal breast cancer. Furthermore, we observed that CD105 expression in CAFs serves as an independent prognostic biomarker for MFS, particularly for BMFS. Additionally, our study demonstrates that the presence of CD105 (+) CAFs is associated with the presence of breast microcalcifications. While further investigation is needed to fully understand the underlying mechanisms, our findings suggest that CD105 (+) CAFs could be involved in the formation of the bone marrow/bone pre-metastatic niche and, consequently, in the development of BM. The presence of this type of metastasis significantly impacts patients’ quality of life, leading to skeletal-related events and ultimately diminishing survival rates. Therefore, the evaluation of CD105 (+) CAFs could be crucial to stratify BCPs based on their individual risk profile for the development of BM, enhancing treatment strategies and outcomes.

## Data Availability

Publicly available datasets were analyzed in this study. This data can be found here: https://www.cbioportal.org/study/summary?id&equals;brca_metabric.

## Abbreviations

BCPs, breast cancer patients; BM, bone metastasis; BMFS, bone metastasis-free survival; CAFs, cancer-associated fibroblast; ER, estrogen receptor; MFS, metastasis-free survival; mix M, mix metastasis; mix MFS, mix metastasis-free survival; MSCs, mesenchymal stem cells; OS, overall survival; PR, progesterone receptor; RFS, relapse-free survival; VM, visceral metastasis; VMFS, visceral metastasis-free survival.

## References

[B1] Barrios C. H. (2022). Global challenges in breast cancer detection and treatment. Breast 62 (S1), S3–S6. 10.1016/j.breast.2022.02.003 35219542 PMC9097801

[B2] Bitencourt A. Rossi Saccarelli C. Morris E. A. Flynn J. Zhang Z. Khan A. (2020). Regional lymph node involvement among patients with de novo metastatic breast cancer. JAMA Netw. Open 3 (10), e2018790. 10.1001/jamanetworkopen.2020.18790 33034638 PMC7547365

[B3] Bloom H. J. Richardson W. W. (1957). Histological grading and prognosis in breast cancer a study of 1409 cases of which 359 have been followed for 15 years. Br. J. Cancer 11 (3), 359–377. 10.1038/bjc.1957.43 13499785 PMC2073885

[B4] Brierley J. D. Gospodarowicz M. K. Wittekind C. (2017). TNM classification of malignant tumours. 8th edn. 10.1016/S1470-2045(17)30438-2 PMC5851445 28677562

[B5] Cerami E. Gao J. Dogrusoz U. Gross B. E. Sumer S. O. Aksoy B. A. (2012). The cBio cancer genomics portal: an open platform for exploring multidimensional cancer genomics data. Cancer Discov. 2 (5), 401–404. 10.1158/2159-8290.CD-12-0095 22588877 PMC3956037

[B6] Charpin C. Dales J.-P. Garcia S. Carpentier S. Djemli A. Andrac L. (2004). Tumor neoangiogenesis by CD31 and CD105 expression evaluation in breast carcinoma tissue microarrays. Clin. Cancer Res. 10 (17), 5815–5819. 10.1158/1078-0432.CCR-04-0021 15355911

[B7] Cheng B. Yu Q. Wang W. (2023). Intimate communications within the tumor microenvironment: stromal factors function as an orchestra. J. Biomed. Sci. 30 (1), 1. 10.1186/s12929-022-00894-z 36600243 PMC9814473

[B8] Cid S. Eiro N. Fernández B. Sánchez R. Andicoechea A. Fernández-Muñiz P. I. (2017). ‘Prognostic influence of tumor stroma on breast cancer subtypes’. Clin. Breast Cancer 18, e123–e133. [Preprint]. 10.1016/j.clbc.2017.08.008 28927692

[B9] Clemenceau A. Michou L. Diorio C. Durocher F. (2020). Breast cancer and microcalcifications: an osteoimmunological disorder? Int. J. Mol. Sci. 21 (22), 8613–8636. 10.3390/ijms21228613 33203195 PMC7696282

[B10] Conklin M. W. Keely P. J. (2012). Why the stroma matters in breast cancer: insights into breast cancer patient outcomes through the examination of stromal biomarkers. Cell Adhesion Migr. 6 (3), 249–260. 10.4161/cam.20567 PMC3427239 22568982

[B11] Dales J.-P. Garcia S. Andrac L. Carpentier S. Ramuz O. Lavaut M.-N. (2004). Prognostic significance of angiogenesis evaluated by CD105 expression compared to CD31 in 905 breast carcinomas: correlation with long-term patient outcome. Int. J. Oncol. 24 (5), 1197–1204. 10.3892/ijo.24.5.1197 15067342

[B12] de Kruijf E. M. van Nes J. G. H. van de Velde C. J. H. Putter H. Smit V. T. H. B. M. Liefers G. J. (2011). Tumor–stroma ratio in the primary tumor is a prognostic factor in early breast cancer patients, especially in triple-negative carcinoma patients. Breast Cancer Res. Treat. 125 (3), 687–696. 10.1007/s10549-010-0855-6 20361254

[B13] de Visser K. E. Joyce J. A. (2023). The evolving tumor microenvironment: from cancer initiation to metastatic outgrowth. Cancer Cell 41 (3), 374–403. 10.1016/j.ccell.2023.02.016 36917948

[B14] Dittmer J. Oerlecke I. Leyh B. (2011). “Involvement of mesenchymal stem cells in breast cancer progression,” in Breast cancer - focusing tumor microenvironment, stem cells and metastasis (InTech). 10.5772/21325

[B15] Dwyer R. M. Potter-Beirne S. M. Harrington K. A. Lowery A. J. Hennessy E. Murphy J. M. (2007). Monocyte chemotactic protein-1 secreted by primary breast tumors stimulates migration of mesenchymal stem cells. Clin. Cancer Res. 13 (17), 5020–5027. 10.1158/1078-0432.CCR-07-0731 17785552

[B16] Elwakeel E. Weigert A. (2021). Breast cancer CAFs: spectrum of phenotypes and promising targeting avenues. Int. J. Mol. Sci. 22 (21), 11636. 10.3390/ijms222111636 34769066 PMC8583860

[B17] Giorello M. B. Borzone F. R. Labovsky V. Piccioni F. V. Chasseing N. A. (2021). Cancer-associated fibroblasts in the breast tumor microenvironment. J. Mammary Gl. Biol. Neoplasia 26 (2), 135–155. 10.1007/s10911-020-09475-y 33398516

[B18] Heneberg P. (2016). Paracrine tumor signaling induces transdifferentiation of surrounding fibroblasts. Crit. Rev. Oncology/Hematology 97, 303–311. 10.1016/j.critrevonc.2015.09.008 26467073

[B19] Kalluri R. Zeisberg M. (2006). Fibroblasts in cancer. Nat. Rev. Cancer 6 (5), 392–401. 10.1038/nrc1877 16572188

[B20] Karnoub A. E. Dash A. B. Vo A. P. Sullivan A. Brooks M. W. Bell G. W. (2007). Mesenchymal stem cells within tumour stroma promote breast cancer metastasis. Nature 449 (7162), 557–563. 10.1038/nature06188 17914389

[B21] Labovsky V. Martinez L. M. Davies K. M. García-Rivello H. De Luján Calcagno M. Matas A. (2015). Association between ligands and receptors related to the progression of early breast cancer in tumor epithelial and stromal cells. Clin. Breast Cancer 15 (1), e13–e21. 10.1016/j.clbc.2014.05.006 25044301

[B22] Li W. Yang D. Wang S. Guo X. Lang R. Fan Y. (2011). Increased expression of CD146 and microvessel density (MVD) in invasive micropapillary carcinoma of the breast: comparative study with invasive ductal carcinoma-not otherwise specified. Pathology - Res. Pract. 207 (12), 739–746. 10.1016/j.prp.2011.09.009 22051146

[B23] Ma X. Liu C. Xu X. Liu L. Gao C. Zhuang J. (2020). Biomarker expression analysis in different age groups revealed age was a risk factor for breast cancer. J. Cell. Physiology 235 (5), 4268–4278. 10.1002/jcp.29304 31608996

[B24] Martinez L. M. Labovsky V. de Luján Calcagno M. Davies K. M. Rivello H. G. Bianchi M. S. (2015). “CD105 expression on CD34-negative spindle-shaped stromal cells of primary tumor is an unfavorable prognostic marker in early breast cancer patients,”. Editor Serra R., 10, e0121421. 10.1371/journal.pone.0121421 PLOS ONE 3 25803686 PMC4372565

[B25] Meer D. J. Kramer I. Maaren M. C. Diest P. J. Linn S. Maduro J. H. (2021). Comprehensive trends in incidence, treatment, survival and mortality of first primary invasive breast cancer stratified by age, stage and receptor subtype in The Netherlands between 1989 and 2017. Int. J. Cancer 148 (9), 2289–2303. 10.1002/ijc.33417 33252836 PMC8048677

[B26] Mimeault M. Batra S. K. (2014). Altered gene products involved in the malignant reprogramming of cancer stem/progenitor cells and multitargeted therapies. Mol. Aspects Med. 39 (1), 3–32. 10.1016/j.mam.2013.08.001 23994756 PMC3938987

[B27] Muñoz T. G. Amaral A. T. Puerto-Camacho P. Peinado H. de Álava E. (2021). Endoglin in the spotlight to treat cancer. Int. J. Mol. Sci., 1–25. 10.3390/ijms22063186 33804796 PMC8003971

[B28] Olfatbakhsh A. Heidari L. Omidi Z. Hashemi E.-S. Ansari M. Mozaffarian S. (2022). Long-term survival and prognostic factors of breast cancer. Archives Iran. Med. 25 (9), 609–616. 10.34172/aim.2022.96 PMC10685763 37543886

[B29] Othman A. Winogradzki M. Lee L. Tandon M. Blank A. Pratap J. (2021). Bone metastatic breast cancer: advances in cell signaling and autophagy related mechanisms. Cancers 13 (17), 4310. 10.3390/cancers13174310 34503118 PMC8431094

[B30] Palafox M. Ferrer I. Pellegrini P. Vila S. Hernandez-Ortega S. Urruticoechea A. (2012). RANK induces epithelial-mesenchymal transition and stemness in human mammary epithelial cells and promotes tumorigenesis and metastasis. Cancer Res. 72 (11), 2879–2888. 10.1158/0008-5472.CAN-12-0044 22496457

[B31] Pasanen I. Lehtonen S. Sormunen R. Skarp S. Lehtilahti E. Pietilä M. (2016). Breast cancer carcinoma-associated fibroblasts differ from breast fibroblasts in immunological and extracellular matrix regulating pathways. Exp. Cell Res. 344 (1), 53–66. 10.1016/j.yexcr.2016.04.016 27112989

[B32] Pestalozzi B. C. Luporsi-Gely E. Jost L. M. Bergh J. ESMO Guidelines Task Force (2005). ESMO Minimum Clinical Recommendations for diagnosis, adjuvant treatment and follow-up of primary breast cancer. Ann. Oncol. 16 (1), i7–i9. 10.1093/annonc/mdi825 15888763

[B33] Potter S. M. Dwyer R. M. Hartmann M. C. Khan S. Boyle M. P. Curran C. E. (2012). Influence of stromal–epithelial interactions on breast cancer *in vitro* and *in vivo* . Breast Cancer Res. Treat. 131 (2), 401–411. 10.1007/s10549-011-1410-9 21344235

[B34] Rau K. M. Huang C. C. Chiu T. J. Chen Y. Y. Lu C. C. Liu C. T. (2012). Neovascularization evaluated by CD105 correlates well with prognostic factors in breast cancers. Exp. Ther. Med. 4 (2), 231–236. 10.3892/etm.2012.594 23139713 PMC3460296

[B35] Raz Y. Cohen N. Shani O. Bell R. E. Novitskiy S. V. Abramovitz L. (2018). Bone marrow–derived fibroblasts are a functionally distinct stromal cell population in breast cancer. J. Exp. Med. 215 (12), 3075–3093. 10.1084/jem.20180818 30470719 PMC6279405

[B36] Rosen P. P. Saigo P. E. Braun D. W. Weathers E. Fracchia A. A. Kinne D. W. (1981). Axillary micro- and macrometastases in breast cancer: prognostic significance of tumor size. Ann. Surg. 194 (5), 585–591. 10.1097/00000658-198111000-00006 7294929 PMC1345263

[B37] Rudnick J. A. Kuperwasser C. (2012). Stromal biomarkers in breast cancer development and progression. Clin. Exp. Metastasis 29 (7), 663–672. 10.1007/s10585-012-9499-8 22684404

[B38] Ruocco M. R. Avagliano A. Granato G. Imparato V. Masone S. Masullo M. (2018). Involvement of breast cancer-associated fibroblasts in tumor development, therapy resistance and evaluation of potential therapeutic strategies. Curr. Med. Chem. 25 (29), 3414–3434. 10.2174/0929867325666180309120746 29521203

[B39] Saini H. Rahmani Eliato K. Veldhuizen J. Zare A. Allam M. Silva C. (2020). The role of tumor-stroma interactions on desmoplasia and tumorigenicity within a microengineered 3D platform. Biomaterials 247, 119975. 10.1016/j.biomaterials.2020.119975 32278213

[B40] Salimifard S. Masjedi A. Hojjat-Farsangi M. Ghalamfarsa G. Irandoust M. Azizi G. (2020). Cancer associated fibroblasts as novel promising therapeutic targets in breast cancer. Pathology Res. Pract. 216 (5), 152915. 10.1016/j.prp.2020.152915 32146002

[B41] Salvador F. Llorente A. Gomis R. R. (2019). From latency to overt bone metastasis in breast cancer: potential for treatment and prevention. J. Pathology 249 (1), 6–18. 10.1002/path.5292 PMC6771808 31095738

[B42] Sappino A. ‐P. Skalli O. Jackson B. Schürch W. Gabbiani G. (1988). Smooth‐muscle differentiation in stromal cells of malignant and non‐malignant breast tissues. Int. J. Cancer 41 (5), 707–712. 10.1002/ijc.2910410512 2835323

[B43] Sarkar M. Nguyen T. Gundre E. Ogunlusi O. El-Sobky M. Giri B. (2023). Cancer-associated fibroblasts: the chief architect in the tumor microenvironment. Front. Cell Dev. Biol. 11, 1089068–1089124. 10.3389/fcell.2023.1089068 36793444 PMC9923123

[B44] Scimeca M. Giocondo R. Montanaro M. Granaglia A. Bonfiglio R. Tancredi V. (2020). BMP-2 variants in breast epithelial to mesenchymal transition and microcalcifications origin. Cells 9 (6), 1381. 10.3390/cells9061381 32498363 PMC7348762

[B45] Senkus E. Kyriakides S. Penault-Llorca F. Poortmans P. Thompson A. Zackrisson S. (2015). Primary breast cancer: ESMO clinical practice guidelines for diagnosis, treatment and follow-up. Ann. Oncol. 26, 8–30. 10.1093/annonc/mdv298 23970019

[B46] Tan C.-C. Li G.-X. Tan L.-D. Du X. Li X.-Q. He R. (2016). Breast cancer cells obtain an osteomimetic feature via epithelial-mesenchymal transition that have undergone BMP2/RUNX2 signaling pathway induction. Oncotarget 7 (48), 79688–79705. 10.18632/oncotarget.12939 27806311 PMC5346745

[B47] Walker R. A. (2001). The complexities of breast cancer desmoplasia. Breast Cancer Res. 3 (3), 143–145. 10.1186/bcr287 11305947 PMC138677

[B48] Weissenbacher T. M. Zschage M. Janni W. Jeschke U. Dimpfl T. Mayr D. (2010). Multicentric and multifocal versus unifocal breast cancer: is the tumor-node-metastasis classification justified? Breast Cancer Res. Treat. 122 (1), 27–34. 10.1007/s10549-010-0917-9 20454925

[B49] Wernicke M. Piñeiro L. C. Caramutti D. Dorn V. G. Raffo M. M. L. Guixa H. G. (2003). Breast cancer stromal myxoid changes are associated with tumor invasion and metastasis: A central role for hyaluronan. Mod. Pathol. 16 (2), 99–107. 10.1097/01.MP.0000051582.75890.2D 12591961

[B50] Wernicke M. Roitman P. Manfre D. Stern R. (2011). Breast cancer and the stromal factor. The “prometastatic healing process” hypothesis. Medicina (B Aires) 71 (1), 15–21. 21296715

[B51] Yamauchi M. Barker T. H. Gibbons D. L. Kurie J. M. (2018). The fibrotic tumor stroma. J. Clin. Investigation 128 (1), 16–25. 10.1172/JCI93554 PMC5749516 29293090

[B52] Zeltz C. Primac I. Erusappan P. Alam J. Noel A. Gullberg D. (2020). Cancer-associated fibroblasts in desmoplastic tumors: emerging role of integrins. Seminars Cancer Biol. 62 (5009), 166–181. 10.1016/j.semcancer.2019.08.004 31415910

