# Supplementary material for: CD105 expression in cancer-associated fibroblasts: a biomarker for bone metastasis in early invasive ductal breast cancer patients
Source: Front Cell Dev Biol. 2023 Aug 31;11:1250869. doi: 10.3389/fcell.2023.1250869 (PMC10501720; doi:10.3389/fcell.2023.1250869)
Supplement: Supplementary file 1 [file Table1.DOCX]

**Supplementary table 1.**

| ***Characteristics of the breast tumor stroma*** | ***n*** | ***% Intratumoral Stroma*** | | ***% Fibroblast*** | | ***Collagen deposition*** | | ***Lymphatic infiltration*** | | ***Desmoplasia*** | | ***Mixoid changes*** | | ***Blood vascularization*** | | ***Lymphatic vascularization*** | |
| --- | --- | --- | --- | --- | --- | --- | --- | --- | --- | --- | --- | --- | --- | --- | --- | --- | --- |
|  |  | ***> 50*** | ***p*** | ***Large amount*** | ***p*** | ***Large amount*** | ***p*** | ***Large amount*** | ***p*** | ***Large amount*** | ***p*** | ***Large amount*** | ***p*** | ***Large amount*** | ***p*** | ***Large amount*** | ***p*** |
| ***Age (years)*** | | | | | | | | | | | | | |  |  |  |  |
| *< 50* | 89 | 39 | 0.901 | 44 | 0.902 | 19 | 0.059 | 44 | 0.805 | 37 | 0.747 | 3 | 0.103 | 24 | 0.870 | 36 | 0.599 |
| *≥ 50* | 253 | 108 |  | 128 |  | 81 |  | 120 |  | 108 |  | 22 |  | 68 |  | 99 |  |
| ***Tumor size (cm)*** | | | | | | | | | | | | | |  |  |  |  |
| ≤2 | 218 | 98 | 0.364 | 110 | 0.911 | 62 | 0.711 | 104 | 0.911 | 90 | 1.000 | 18 | 0.398 | 52 | 0.188 | 76 | **0.028*** |
| *>2* | 124 | 49 |  | 62 |  | 38 |  | 60 |  | 55 |  | 7 |  | 40 |  | 59 |  |
| ***ER*** | | | | | | | | | | | | | | | | | |
| Negative | 44 | 21 | 0.517 | 20 | 0.521 | 8 | 0.109 | 31 | **0.002** | 15 | 0.219 | 2 | 0.755 | 9 | 0.655 | 20 | **0.015** |
| Positive | 298 | 126 |  | 152 |  | 92 |  | 133 |  | 130 |  | 23 |  | 83 |  | 115 |  |
| ***PR*** | | | | | | | | | | | | | | | | | |
| Negative | 59 | 23 | 0.564 | 24 | 0.116 | 12 | 0.116 | 36 | **0.032** | 19 | 0.668 | 4 | 1.000 | 16 | 0.432 | 27 | **0.005** |
| Positive | 283 | 124 |  | 148 |  | 88 |  | 128 |  | 126 |  | 21 |  | 76 |  | 108 |  |
| ***Her2/neu*** | | | | | | | | | | | | | | | | | |
| Negative | 283 | 119 | 0.665 | 140 | 0.666 | 81 | 0.638 | 130 | 0.198 | 123 | 0.412 | 22 | 0.591 | 76 | 0.322 | 110 | 0.084 |
| Positive | 59 | 27 |  | 31 |  | 19 |  | 33 |  | 21 |  | 3 |  | 16 |  | 24 |  |
| ***Histological grade*** | | | | | | | | | | | | | | | | | |
| *G1* | 35 | 21 | **0.024** | 20 | 0.266 | 14 | **0.012** | 14 | 0.1110 | 14 | 0.092 | 4 | 0.134 | 6 | 0.377 | 12 | 0.438 |
| *G2* | 182 | 82 |  | 96 |  | 61 |  | 81 |  | 90 |  | 16 |  | 50 |  | 70 |  |
| *G3* | 125 | 44 |  | 56 |  | 25 |  | 69 |  | 41 |  | 5 |  | 36 |  | 53 |  |
| ***Regional lymph nodes*** | | | | | | | | | | | | | | | | | |
| Negative | 234 | 97 | 0.413 | 116 | 0.729 | 74 | 0.162 | 110 | 0.642 | 98 | 0.881 | 17 | 1.000 | 65 | 0.544 | 90 | 0.414 |
| Positive | 108 | 50 |  | 56 |  | 26 |  | 54 |  | 47 |  | 8 |  | 27 |  | 45 |  |
| ***Local relapse*** | | | | | | | | | | | | | | | | | |
| Negative | 302 | 130 | 1.000 | 152 | 1.000 | 92 | 0.199 | 141 | 0.239 | 130 | 0.635 | 22 | 1.000 | 83 | 0.820 | 120 | 0.616 |
| Positive | 40 | 17 |  | 20 |  | 8 |  | 23 |  | 15 |  | 3 |  | 9 |  | 15 |  |
| ***Metastatic occurrence*** | | | | | | | | | | | | | | | | | |
| Negative | 265 | 108 | 0.150 | 132 | 0.797 | 78 | 1.000 | 121 | 0.122 | 119 | 0.168 | 22 | 0.316 | 77 | 0.287 | 105 | 0.250 |
| Positive | 77 | 39 |  | 40 |  | 22 |  | 43 |  | 26 |  | 3 |  | 15 |  | 30 |  |
| ***Bone metastatic occurrence*** | | | | | | | | | | | | | | | | | |
| Negative | 313 | 130 | 0.081 | 154 | 0.244 | 93 | 0.671 | 142 | 0.002 | 133 | 1.000 | 24 | 0.708 | 85 | 1.000 | 120 | **0.024** |
| Positive | 29 | 17 |  | 18 |  | 7 |  | 22 |  | 12 |  | 1 |  | 7 |  | 15 |  |
| ***Visceral metastatic occurrence*** | | | | | | | | | | | | | | | | | |
| Negative | 302 | 128 | 0.611 | 153 | 0.738 | 88 | 1.000 | 144 | 0.867 | 134 | 0.155 | 24 | 0.335 | 84 | 0.273 | 120 | 1.000 |
| Positive | 40 | 19 |  | 19 |  | 12 |  | 20 |  | 11 |  | 1 |  | 8 |  | 15 |  |
| ***Mix metastatic occurrence*** | | | | | | | | | | | | | | | | | |
| Negative | 334 | 144 | 1.000 | 169 | 0.499 | 97 | 0.696 | 163 | 0.069 | 142 | 1.000 | 24 | 0.461 | 92 | 1.000 | 135 | 0.318 |
| Positive | 8 | 3 |  | 3 |  | 3 |  | 1 |  | 3 |  | 1 |  | 0 |  | 0 |  |

**Supplementary Table 1.** Association between stromal histological features and classical prognostic markers, as well as local relapse, metastatic recurrence, bone metastatic recurrence, visceral metastatic recurrence and mix metastatic recurrence in a cohort of 342 untreated early breast cancer patients. Fisher's exact test was used for the association between variables, * p-value <0.050. ER *estrogen receptor*, PR *progesterone receptor*
